# Whole genome assembly of a natto production strain Bacillus subtilis natto from very short read data

**DOI:** 10.1186/1471-2164-11-243

**Published:** 2010-04-16

**Authors:** Yukari Nishito, Yasunori Osana, Tsuyoshi Hachiya, Kris Popendorf, Atsushi Toyoda, Asao Fujiyama, Mitsuhiro Itaya, Yasubumi Sakakibara

**Affiliations:** 1 Department of Biosciences and Informatics, Keio University, Hiyoshi, Kohoku-ku, Yokohama, Japan; 2 Department of Computer and Information Science, Seikei University, Musashino, Tokyo, Japan; 3 Center for Genetic Resource Information, National Institute of Genetics, Shizouka, Japan; 4 Principles of Informatics Research Division, National Institute of Informatics, Tokyo, Japan; 5 Institute for Advanced Biosciences, Keio University, Minato, Tokyo, Japan

## Abstract

**Background:**

*Bacillus subtilis *natto is closely related to the laboratory standard strain *B. subtilis *Marburg 168, and functions as a starter for the production of the traditional Japanese food "natto" made from soybeans. Although re-sequencing whole genomes of several laboratory domesticated *B. subtilis *168 derivatives has already been attempted using short read sequencing data, the assembly of the whole genome sequence of a closely related strain, *B. subtilis *natto, from very short read data is more challenging, particularly with our aim to assemble one fully connected scaffold from short reads around 35 bp in length.

**Results:**

We applied a comparative genome assembly method, which combines *de novo *assembly and reference guided assembly, to one of the *B. subtilis *natto strains. We successfully assembled 28 scaffolds and managed to avoid substantial fragmentation. Completion of the assembly through long PCR experiments resulted in one connected scaffold for *B. subtilis *natto. Based on the assembled genome sequence, our orthologous gene analysis between natto BEST195 and Marburg 168 revealed that 82.4% of 4375 predicted genes in BEST195 are one-to-one orthologous to genes in 168, with two genes in-paralog, 3.2% are deleted in 168, 14.3% are inserted in BEST195, and 5.9% of genes present in 168 are deleted in BEST195. The natto genome contains the same alleles in the promoter region of *degQ *and the coding region of *swrAA *as the wild strain, RO-FF-1.

These are specific for γ-PGA production ability, which is related to natto production. Further, the *B. subtilis *natto strain completely lacked a polyketide synthesis operon, disrupted the plipastatin production operon, and possesses previously unidentified transposases.

**Conclusions:**

The determination of the whole genome sequence of *Bacillus subtilis *natto provided detailed analyses of a set of genes related to natto production, demonstrating the number and locations of insertion sequences that *B. subtilis *natto harbors but *B. subtilis *168 lacks. Multiple genome-level comparisons among five closely related *Bacillus *species were also carried out. The determined genome sequence of *B. subtilis *natto and gene annotations are available from the Natto genome browser http://natto-genome.org/.

## Background

Recent significant progress in short read sequencing and computer technologies that can handle large volumes of short read data using high-speed CPUs with increased memory has enabled the assembly and determination of a bacterial genome in single laboratories. Using these technologies, several attempts have been made to determine various bacterial genomes such as those of *Helicobacter acinonychis *[1], *Staphylococcus aureus *[2], and *Bacillus subtilis *laboratory strains [3,4]. There are fundamentally two different approaches for assembling bacterial genomes from short read data [5], namely, *de novo *assembly and reference guided assembly (re-sequencing or mapping to a reference genome). In this paper, we apply our assembly pipeline that combines *de novo *assembly and reference guided assembly in order to determine the *B. subtilis *natto genome sequence.

The first genome sequence of the *B. subtilis *strain Marburg 168 [6], the best characterized Gram-positive bacterium, provided a great gain to microbiology research. Although several derivatives of *B. subtilis *168 have recently been sequenced using SRS data [3], domesticated strains propagated in the laboratory over time lack some traits of the original strain, such as insertion sequences, plasmids [7], and the ability to produce γ-PGA and hence mucoid biofilm formation [8].

The traditional Japanese food natto (fermented soybean) is made from soybeans fermented without salt by the bacterium *B. subtilis *natto starter strain (see Additional file 1, Figure S1 for a simple experiment demonstrating natto fermentation). At least three kinds of commercial natto starter strains are available in Japan. They are classified as *B. subtilis *natto closely related to the laboratory strain *B. subtilis *Marburg 168, which has about 4,100 protein-encoding genes in a 4,215 kbp genome [4,6]. Natto is an ideal food because it can be easily prepared, it has a complete set of nutrients, and it can be stored via its production of fungicidal antibiotics [9]. Many studies have attempted to describe the complex process by which natto is produced, a process that can be divided into several steps, including secretion of proteases on the surface of soybeans, incorporation of digested amino acids, and synthesis of *γ*-poly-DL-glutamic acid (γ-PGA), the major constituent of a viscous biofilm [10]. Furthermore, γ-PGA was identified as an extracellular polymer that can enhance biofilm formation [8].

Extensive biochemical and molecular genetic studies have been conducted on the genes and enzymes involved in natto fermentation [9,10]. A limited number of genetically characterized gene homologues, such as *pgsBCA *(*ywsC*-*ywtAB *in the Marburg 168 counterpart) [9,10], *degQ *(*iep*) [11] and *comQXPA *[12] are also present in the genome of *B. subtilis *Marburg 168. This laboratory strain does not produce capsular PGA, suggesting that highly coordinated regulation of both gene expression and physiological conditions during growth on the surface of soybeans is required for high-quality natto starter strains.

Recently, it has been revealed that the inability of the laboratory strain JH642 of *B. subtilis *to produce γ-PGA was due to two nucleotide changes [8]: (a) a single nucleotide substitution in the promoter region of *degQ*; and (b) a single nucleotide insertion in the coding region of *swrAA*. The introduction of the *degQ *and *swrAA *alleles from a wild *B. subtilis *strain RO-FF-1 (isolated from the Mojave desert) into the *B. subtilis *JH642 strain was necessary and sufficient to allow γ-PGA production and consequent formation of a mucoid colony phenotype. We confirmed that the *B. subtilis *natto genome sequence determined in this study contains the same alleles in the promoter region of *degQ *and the coding region of *swrAA *as the strain RO-FF-1. Therefore, this natto strain does not contain the thymine-to-cytosine nucleotide substitution in the *degQ *promoter region, and the single adenine nucleotide insertion in the coding region of *swrAA*, which induced the pseudogenization of *swrAA *in 168 strain according to the latest annotation for the updated release of 168 genome [4].

We conducted a multiple genome comparison among five closely related *Bacillus *species including the *B. subtilis *natto sequence determined in this study. It was revealed that there were many insertions and deletions but no significant rearrangements, and gene orders were well conserved among the five genomes with two large syntenic segments detected. Furthermore, in the operon structure of the four quorum-sensing genes *comQ*, *comX*, *comP *and *comA*, our natto genome sequence revealed that the region of DNA starting at the 5' end of the coding sequence of *comQ *and ending at the middle of the coding region of *comP *via *comX *was significantly divergent and contained almost no sequence similarities to *B. subtilis *Marburg 168, as previously observed in *B. subtilis *natto NAF4 strain [12]. The amino acid sequence of ComX, containing a pheromone peptide, is identical for the two natto strains BEST195 and NAF4. Together with the fact that ComP and ComA were identified as regulators of biofilm formation along with the DegSU, DegQ and SwrA regulators of γ-PGA production [8], these observations are consistent with the interpretation that the *comQXP *gene module determines a *B. subtilis *natto-specific cell density signaling system.

## Results

### Mapping short read sequence data to the reference genome

Genomic DNA was extracted from *B. subtilis *natto BEST195 [13] and whole genome shotgun sequences were obtained using the Illumina genome analyzer. A total of 15,296,102 paired-end reads of 36 bp length were generated, with the average length of inserts of paired-end reads at 163 bp. To control each step in the following experiments, we re-sequenced *B. subtilis *Marburg 168 (1A1) [4,6] (The sequencing results for strain 168 are provided in Additional file 2, Data S1).

The generated paired-end reads were mapped to the published *B. subtilis *reference genome of Marburg 168 using Mapping and Assembly with Qualities (MAQ) software [14]. Of the total reads, 79.1% could be mapped to the reference genome with 131-fold sequencing coverage across the entire genome. This fold coverage rate is in the mid range between an extremely high level of coverage (285 times) used for *de novo *assembly of a *Helicobacter acinonychis *genome [1] and a low level of coverage (48 times) used for the assembly of a *Staphylococcus aureus *sequence [2].

At each base, a quality score was statistically calculated based on the reads using MAQ. The quality scores describe the confidence that the base is correctly called. A total of 84.6% of all mapped bases had a quality score of 40 or higher, and 84.7% of all the mapped bases had a quality score of 30 or higher. The results are summarized in Table 1. A quality score of 40 was used as the cut-off value, and a consensus sequence was finally produced from the MAQ alignment of short reads of BEST195 against Marburg 168 reference genome. We called this consensus sequence the *reference-guided draft*.

**Table 1:** Summary of sequence reads, coverage, and quality score.

Strain	Genomic DNA	Total reads	Mapped reads	Coverage	Quality score 40
	(ng/*μ*l)	(million)	(%)	(fold)	(%)
BEST 195	207	15.3	79.1	131	86.3
Marburg 168	140	13.2	92.2	111	99.7

Genomic DNA was extracted from *B. subtilis natto *BEST195 isolated from Miyagino-based natto, and *B. subtilis *Marburg 168 (1A1). The short paired-end reads were generated by the Illumina genome analyzer GA2. The generated paired-end reads were mapped to the Marburg 168 reference genome using MAQ software. At each base, a quality score was statistically calculated based on the reads using MAQ. The quality scores describe the confidence that the base is correctly called.

### De novo assembly from short read data and sorting generated scaffolds

All short paired-end reads generated by the Illumina genome analyzer were input into the *de novo *assembly software Velvet [15]. A total of 390 scaffolds (scaffold contigs) with an average length of 6,693 bp were produced for *B. subtilis *natto BEST195 with a predefinedcut-off rate. The total size of the produced scaffolds was 4,178 kbp and that of 117 scaffolds with length greater than 1 kbp is 4,138 kbp, that is, 99.0% of all the scaffolds we used for the next step of sorting scaffolds. All statistics regarding the generated short reads and the produced scaffolds are summarized in Table 2. N50 scaffold sizes shown in Table 2 indicate the increased difficulty of *de novo *assembly of BEST195 compared to strain 168. This was mainly due to the presence of insertion sequences in BEST195, which strain 168 lacks.

**Table 2:** Summary of scaffolds produced by Velvet and their sorting.

Strain	Number of total scaffolds	Average length	Total length	N50 scaffold size (bp)	Number of scaffolds longer	Number of aligned scaffolds
		(bp)	(kbp)		than 1 kbp	
BEST 195	390	6,693	4,178	72,513	117	84
Marburg 168	205	11,319	4,222	489,616	45	45

All short paired-end reads generated by the Illumina GA2 were input into the *de novo *assembly software Velvet and a set of scaffolds produced. The scaffolds greater than 1 kbp were aligned to the Marburg 168 reference genome. N50 indicates the scaffold length such that 50% of the *de novo *assembled sequences lies in scaffolds of this size or larger.

**Figure 1:**
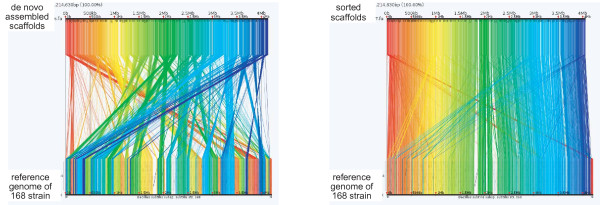
**Sorting scaffolds along Marburg 168 reference genome**. The *de novo *assembled scaffolds were sorted using anchors along the strain Marburg 168 genome and aligned to the Marburg 168 reference genome. Anchors, which are short well-conserved subsequences between each scaffold and the reference genome, were calculated using Murasaki, a multiple genome comparison program [16]. (Left, before sorting) Link plot between unsorted scaffolds and the Marburg 168 reference genome. (Right, after sorting) Link plot between sorted scaffolds and the reference genome. Each line between the scaffolds (upper part of the link plot) and the reference genome (lower) indicates an anchor between them.

Scaffolds greater than 1 kbp were sorted using anchors along the Marburg 168 genome and aligned to the Marburg 168 reference genome. Anchors, which are short, well-conserved subsequences between each scaffold and the reference genome, are calculated using Murasaki, a multiple genome comparison program [16]. The link plot between unsorted scaffolds and the Marburg 168 reference genome and the link plot between sorted scaffolds and the reference genome are displayed in Figure 1. Of the 117 scaffolds greater than 1 kbp, 84 contained anchors of the Marburg 168 genome, and the remaining 33 scaffolds displayed no similarity to the Marburg genome. One scaffold of the remaining 33 was identified as a plasmid with the same sequence as that of pTA1015 [17]. The 84 scaffolds were sorted and placed at the corresponding locations according to anchors along the Marburg 168 genome. Our annotation shows that the unsorted scaffolds contain insertion sequences, transposons, phages and non-coding RNA sequences.

### Combination of two assembly results and completion of the assembly by PCR

We combined the *de novo *assembly with the reference-guided draft to fill the gaps among *de novo *assembled scaffolds using the following three steps.

**Figure 2:**
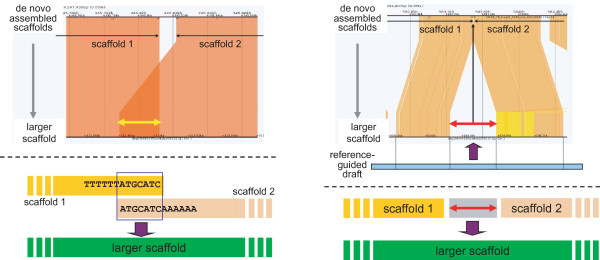
**Concatenating sorted scaffolds to constitute scaffolds**. The *de novo *assembled scaffolds were combined with the reference-guided draft to fill the gaps among the scaffolds. (Left) Two adjacent scaffolds that overlapped were merged into one larger scaffold. (Right) If a subsequence was inserted in the reference-guided draft between two adjacent scaffolds, such scaffolds and the inserted subsequence were concatenated into one scaffold.

Sorted scaffolds were concatenated by aligning to the reference-guided draft using the fast anchor finding algorithm Murasaki [16] and assembled into large scaffolds as follows: (i) Two adjacent scaffolds that overlapped were merged into one larger scaffold (Figure 2 (left)); 41 gaps were filled in this manner. (ii) If a subsequence was inserted in the reference-guided draft between two adjacent scaffolds, such scaffolds and the inserted subsequence were concatenated into one scaffold (Figure 2 (right)); 17 gaps were filled in this manner.

(iii) The remaining gaps were experimentally determined by PCR amplification. For both ends of each scaffold, the specific primers were designed and the gap regions amplified. The size of the PCR products were estimated by gel electrophoresis, enabling determination of the length of all remaining 26 gaps. Most of these products were successfully sequenced using a Sanger sequencer (ABI 3100 Genetic Analyzer).

The PCR experiments confirmed the correct order of all sorted scaffolds that were calculated by alignment to the Marburg 168 strain and the concatenations of scaffolds using the above-mentioned steps (i) and (ii). By using these three steps, one large scaffold (the final draft) was finally constructed from 84 scaffolds. The 32 unplaced scaffolds greater than 1 kbp fitted into the remaining gaps.

The 273 scaffolds less than 1 kbp were also analyzed against the final draft, and of these scaffolds, 169 were found in the draft as subsequences.

All Solexa reads have been deposited in the Read Archive at DDBJ http://www.ddbj.nig.ac.jp/ with accession number DRA000001, and the final genome sequence and annotation have been deposited in DDBJ with accession number AP011541 and AP011542.

### SfiI physical map

**Figure 3:**
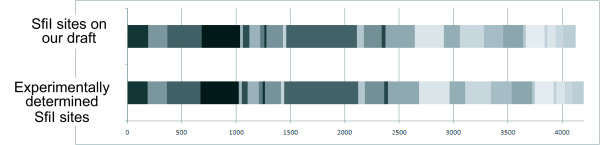
**SfiI physical maps**. Comparison of SfiI physical (restriction site) maps between our draft and the experimental construction [18]. Each block with a different graded gray color indicates an SfiI fragment which must be digested at SfiI restriction site. The number of SfiI restriction sites in both maps is identical and SfiI fragments are similar in sizes within experimental errors, therefore proving the accuracy of our draft.

A detailed SfiI restriction map was experimentally constructed for BEST195 [18]. We compared the SfiI restriction sites between our draft and the experimental physical map as indicated in Figure 3. The number of SfiI restriction sites in both maps was identical and SfiI fragments were similar in size within experimental errors, approximately ± 3%, except three large ones. In particular, the differences in size of fragments containing large gaps at the coordinates 1849008, 3248194, and 3380048 between our draft and the experimental physical map strongly indicate that some undetermined scaffolds, approximately 24 kb in sum, might be included in these remaining gaps.

### Natto production genes

**Figure 4:**
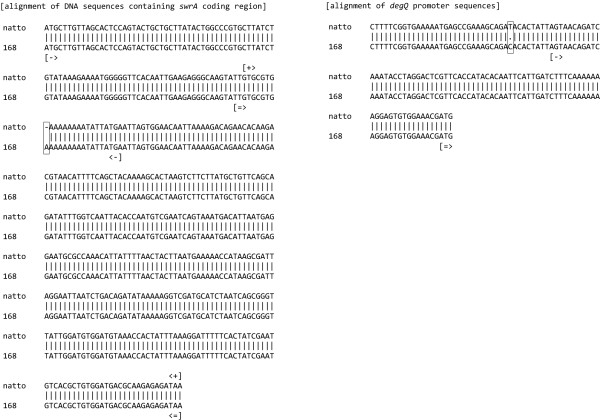
**Analyses of γ-PGA production genes for soybean fermentation**. We confirmed the two nucleotide changes in regions related to γ-PGA production. In the 168 strain, incapable of producing γ-PGA, a single nucleotide is substituted from cytosine to thymine in the promoter region of *degQ *and a single adenine is inserted into the coding region of *swrAA*. These two nucleotide substitutions from 168 strain are specifically present in the natto BEST195 genome. (Left) Alignment between the *swrAA *coding region of natto BEST195 and Marburg 168. The box indicates a single adenine nucleotide insertion position. The pair " [->" and "<-]" indicates the region of the first pseudogene annotation, the pair " [= >" and "< = ]" indicates the region of the second pseudogene annotation in 168 strain, and the pair " [+>" and "<+]" indicates the ORF of *swrAA *coding region in natto BEST195. (Right) Alignment of the *degQ *promoter regions from strains BEST195 and 168. The box indicates the thymine-to-cytosine nucleotide substitution, "[->" indicates the transcription start site, and "[= >" denotes the translation start codon ATG.

Several genes required for soybean fermentation have been of central interest in the genetic, biochemical, and physiological investigation of *B. subtilis *natto. Since γ-PGA was identified as an extracellular polymer that can enhance biofilm formation, and the ability of a wild *B. subtilis *strain RO-FF-1 to produce γ-PGA was due to two nucleotide substitutions [8] from the 168 strain, we confirmed these nucleotide substitutions in our genome draft of *B. subtilis *natto (BEST195) strain. In the 168 strain that is incapable of producing γ-PGA, a single nucleotide was substituted from cytosine to thymine in the promoter region of *degQ*, and a single adenine was inserted into the coding region of *swrAA*. Figure 4 clearly demonstrates that two nucleotide substitutions from the 168 strain are present in the natto BEST195 genome. The alignment of DNA sequences containing the *swrAA *coding region between natto BEST195 and Marburg 168 is shown in Figure 4 (left). It clearly reveals that a single nucleotide insertion in 168 strain broke down the open reading frame (ORF) of *swrAA *and induced the pseudogenization of *swrAA *in 168 strain, which was previously shown to result in the production of a non-functional allele of *swrAA *[19]. The thymine-to-cytosine nucleotide change in the *degQ *promoter region was also observed in the alignment between the *degQ *promoter regions of strains BEST195 and 168 (Figure 4 (right)), and the position of the nucleotide change corresponds to the -10 binding site. Previous work [8] has revealed that the promoter with a cytosine-to-thymine nucleotide change increased the transcription of *degQ *and led to the formation of a mucoid colony morphology.

**Figure 5:**
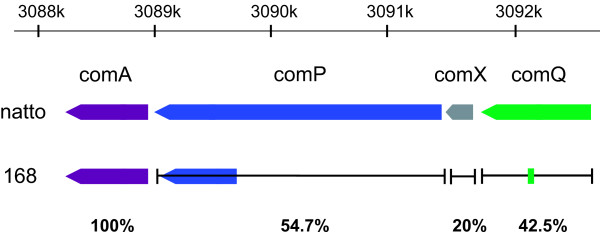
**Analyses of quorum-sensing genes for soybean fermentation**. Large variations in the four quorum-sensing genes *comQ*, *comX*, *comP*, and *comA *were observed between natto BEST195 and 168. The partial broad arrow in strain 168 indicates the identical portion of DNA sequences encoding each gene of the natto strain. The percentages indicate identity of protein sequences between natto BEST195 and 168.

Within the operon structure of the four quorum-sensing genes *comQ*, *comX*, *comP *and *comA*, significant large variation in the DNA region starting at the 5'-end of the coding region of *comQ *and ending in the middle of the coding region of *comP *via *comX *was observed between BEST195 and 168, as shown in Figure 5. This large variation was also previously observed in *B. subtilis *natto NAF4 strain [12].

Interestingly, the amino acid sequences of ComX containing the pheromone peptide between BEST195 and NAF4 are completely identical. These observations are consistent with the interpretation that the *comQXP *gene module determines a *B. subtilis *natto-specific cell density signaling system.

### Plasmid sequences

BEST195 contains two plasmids, pBEST195L and pBEST195S, as described in the Methods section. We have previously revealed that these plasmids are not required for natto production by BEST195 [13]. A 65 kbp plasmid, pLS20, similar to pBEST195L [18,20,21] and pTA1015, similar to pBEST195S [17] have been reported. We screened strains in which both plasmids were absent in order to be able to apply future genetic and molecular cloning works to this *B. subtilis *natto strain. Only the strain missing pBEST195L was obtained and this strain was subjected to the present sequencing. As expected, pBEST195S was shown to be nearly identical to pTA1015.

### Polyketide synthesis gene

**Figure 6:**
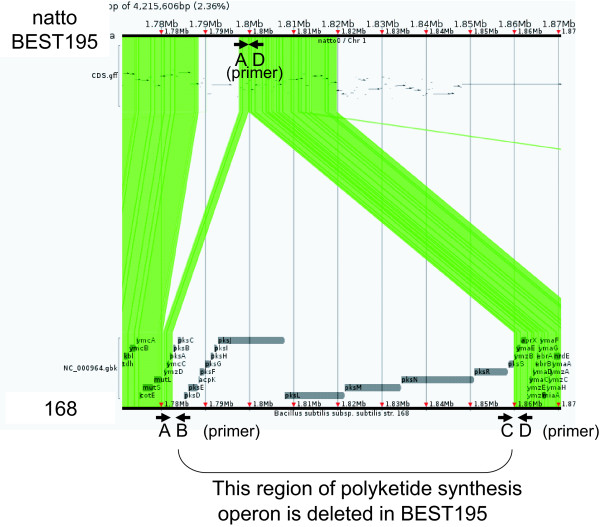
**Polyketide synthesis operon**. An operon structure for a series of polyketide synthesis genes from *pksB *to *pksR *which begins with transcriptional regulator *pksA *and ends with hydroxylase of polyketide *pksS *is completely deleted in *B. subtilis *natto BEST195. Link plot of the alignment between a region including the polyketide synthesis operon in Marburg 168 and the corresponding region in BEST195 is displayed. The alignment was calculated using Murasaki, a multiple genome comparison program [16]. Each line between them indicates an anchor, which is a short well-conserved subsequence. Four PCR primers, A: 5'-AGAAAACAAATTGCAGAAGCAAC-3', B: 5'-GCATGTTGTTAAAGCACATAGCA-3', C: 5'-GATTGCATATGAAGTCACTCGC-3', and D: 5'-TACTCTACTCAGGTTGAGTGGGC-3' are indicated by horizontal short arrows. These primers are designed to amplify both ends of polyketide synthesis operon in 168. The pair of primers A, B and the pair C, D produced the predicted 3.14-kb and 3.10-kb fragments from 168. In contrast, only the pair A, D produced a predicted 1.62-kb fragment from BEST195 (data not shown).

An operon structure for a series of polyketide synthesis genes from *pksB *to *pksR *which begins with transcriptional regulator *pksA *and ends with hydroxylase of polyketide *pksS *is completely deleted in *B. subtilis *natto (Figure 6). Each *B. subtilis *strain, as well as every bacterium, generally contains its own polyketide synthesis operon.

In contrast, only part of genes to synthesize another polyketide, plipastatin, in *B. subtilis *Marburg 168 [22] are present in the *B. subtilis *natto genome. The operon structure of five genes from *ppsA *to *ppsE *in 168 is shown in Additional file 3, Figure S2. Absence of internal *ppsB *and *ppsC *genes in BEST195 suggests that excision via intrachromosomal recombination between two highly similar regions in *ppsA *and *ppsD *occurred. The present partial deletion in BEST 195 is consistent with a similar deletion formation of the plipastatin operon of Marburg 168 recently reported [23].

### Insertion sequence

*B. subtilis *Marburg 168 lacks typical insertion sequences [4,6]. In contrast, many *B. subtilis *natto strains harbor various copies of the insertion sequence (IS) IS*4Bsu*1 [24] and IS*256 *[25]. Our natto draft sequence clearly demonstrated the presence of IS*4Bsu*1 (5 copies) and IS*256 *(6 copies). In addition, we discovered IS*643*-like transposases (pair of orfA and orfB, 3 copies), IS*Bma*2-like transposases (12 copies), IS*Lmo*1-like transposases (pair of orfA and orfB, 11 copies), and several putative transposases. Their locations are summarized in Additional file 4, Table S1. The natto IS is considered to be frequently transposed within the host genome, being consistent with our unpublished observation that the high frequency of IS appearance in BEST195 colonies causes inability to ferment soy proteins. IS-insertion into genes relevant to natto production might be more plausible than spontaneous mutation induced in these genes, since mutation hot regions have not yet been identified in Marburg 168 genomes [3]. Actual transposition activity of those in BEST195 strain remains to be experimentally scrutinized.

### Gene annotation and multiple genome comparison

The gene annotations along with three *Bacillus *species' comparisons are available on our Natto genome browser http://natto-genome.org/, based on the generic genome browser GBrowse [26].

Our orthologous gene analysis using the OASYS program [27] (described in the Methods section in detail) which accurately detects one-to-one orthology relationships between natto BEST195 and Marburg 168 revealed that 82.4% of 4375 predicted genes in BEST195 are one-to-one orthologous to genes in 168, two genes are in-paralog, 3.2% are deleted in 168, 14.3% are inserted in BEST195 (lineage-specific), and 5.9% of genes present in 168 are deleted in BEST195. Further, we calculated comprehensive sequence alignments for those 3610 orthologous genes between *B. subtilis *natto BEST195 and Marburg 168. The list of all the alignments is available in Additional file 5, Data S2, and on the Natto genome browser.

We conducted multiple genome-level comparisons among five closely related *Bacillus *species, Marburg 168, BEST195, *B. amyloliquefaciens *[28], *B. licheniformis *[29], and *B. pumilus *[30]. These five *Bacillus *species exhibited significant genome similarities among all *Bacillus *species genomes. Our multiple genome comparisons revealed that there were numerous insertions and deletions but no significant rearrangements. Gene orders were well conserved among the five genomes and two large syntenic segments, that is defined as a conserved segment descended from a common ancestor without rearrangements, were detected by using the accurate orthology mapping program, OSfinder [31] (described in the Methods section in detail). The link plot of five *Bacillus *species genome comparison, and the dot plot of a pairwise comparison of orthologous genes between BEST195 and Marburg 168, *B. amyloliquefaciens*, *B. licheniformis*, and *B. pumilus *respectively, are displayed in Additional file 6, Figure S3.

## Discussion

There was no single standard *B. subtilis *natto strain similar to *B. subtilis *Marburg 168 whose derivatives have been developed in laboratories worldwide [3,7]. Most information relevant for natto production is deduced from comparative studies in which standard Marburg 168 is employed. Our group has intensively studied BEST195 [13] and the draft sequence determined in this study is consistent with accumulated data reported for other *B. subtilis *natto strains. The present sequence-determined strain BEST195, originally isolated from commercial natto, can become a standard, safe and beneficial *B. subtilis *natto bacterium in terms of a more appropriate host for future applications such as the mass production of useful materials. On the other hand, *B. subtilis *natto BEST195 strain possessed insertion sequences not only expected ones such as IS*4Bsu*1 and IS*256 *but also ones previously unidentified. This is in sharp contrast to *B. subtilis *Marburg 168 that lacks typical insertion sequences. The strain-specific IS feature was observed in our previous study where a *B. subtilis *natto strain BEST217 apparently lacked IS*4Bsu*1 [18]. Together with our present sequence-based conclusion, presence of IS and their population in the genome can draw attention on plausible gene regulations for maintenance and exclusion of IS.

### Validation of our genome assembly method from short read data

Our assembly pipeline (described in the method section in detail) used for determining the BEST195 genome sequence was validated in two manners.

1. A draft sequence using our short read re-sequence data of Marburg 168 and the previous release [6] of 168 genome sequence as reference was assembled by our assembly method. The assembled sequence was compared with the updated release [4] of 168 genome sequence by using BLASTZ, a whole genome alignment program [32], to see how many bases are matched.

2. The generated short read data from BEST195 was divided into two subsets, a draft sequence was assembled using one subset by our assembly method, and short reads in the other subset were mapped into the assembled sequence to see how many reads can be mapped.

The result for the validation test (1) is that the rate of mismatch in the alignment between the assembled sequence by our method and the updated release of 168 genome is 0.21% in our draft sequence and 0.17% in the updated release of 168 while the rate of mismatch between the previous release and the updated release of 168 genome is 0.37% in the previous release and 0.39% in the updated release. Thus, our draft sequence improved the previous release of 168 genome. On the other hand, the result for the test (2) is that 96.57% of reads in one subset were mapped to the draft sequence assembled from reads in the other subset. These two validation results demonstrated the proper reliability of our assembly method and an adequate quality of our genome draft, while the Sfil profile differences between restriction site maps could still leave a possibility of some misassemblies in our draft.

### SRS ability to detect SNPs and large variation

First, the precise identification of a single nucleotide substitution in the promoter region of *degQ *and a single nucleotide insertion in the coding region of *swrAA *between the 168 and BEST195 strains confirmed the ability of SRS technology to detect SNPs. Second, our assembly pipeline that combines *de novo *assembly and reference guided assembly was proven to be capable of detecting large variations in DNA region starting at the 5'-end of the *comQ *coding region and ending in the middle of the coding region of *comA *via *comP*. A simple mapping method that maps the generated short read data onto a published reference genome cannot cover the species-specific regions divergent from a reference genome. Third, our assembly pipeline also succeeded in determining the complete deletion of an operon structure of polyketide synthesis genes, as well as many insertions of IS copies such as IS*4Bsu*1. Fourth, our assembly pipeline succeeded in simultaneously assembling an additional *B. subtilis *specific plasmid sequence.

### Assembly limitation using SRS data: analysis of scaffold ends

We conducted sequence analyses and annotations for both ends of all scaffolds greater than 1 kbp in order to clarify the reason why *de novo *assembly terminated at the positions by the Velvet assembler.

**Figure 7:**
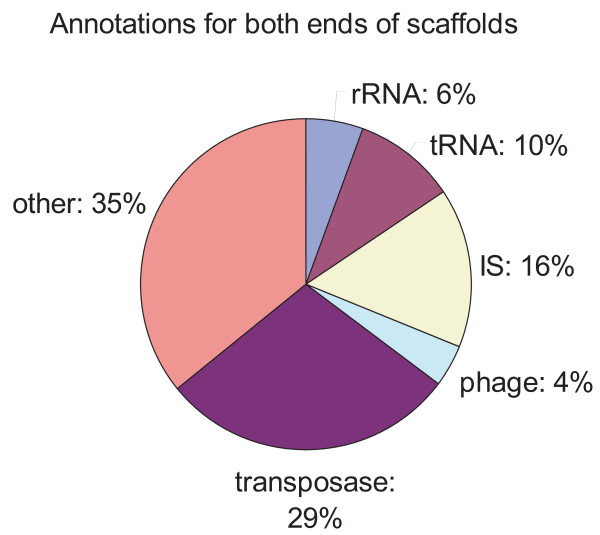
**Analyses of scaffold ends that the Velvet assembler generated**. Annotations for both ends of all scaffolds of length greater than 1 kbp revealed that *de novo *assembly by the Velvet assembler terminated at repeat sequences such as tRNAs, ISs, and phages.

As illustrated in Figure 7, about 70% of the ends of all scaffolds generated by the Velvet assembler are repeat sequences such as tRNAs, ISs and phages. Although strain 168 is known to have no ISs and is therefore easier to assemble than BEST195, the previous attempt to assemble several laboratory 168 derivatives using SRS [3] could not be completed with one connected scaffold. This indicates the difficulty and limitation of genome assembly using short read sequence data to span across longer repeat sequences such as insertion sequences, transposons, and non-coding RNA sequences. More systematic analyses regarding the limitations of *de novo *assembly from short read data using various short read assemblers have been done in an experiment involving the *Pseudomonas syringae *genome assembly [33], and also reported in a technical note on the Illumina website.

## Conclusions

Our research provided two distinguished features: a short-read assembly pipeline that combines *de novo *assembly and reference guided assembly, and determination of the whole genome sequence of *Bacillus subtilis *natto with detailed analysis of a set of genes related to natto production. Using a short-read assembly pipeline and PCR experiments to determine the remaining gaps, one large scaffold (the final draft) was finally constructed. The usefulness of our genome assembly method was proven in terms of single polynucleotide polymorphism (SNP) detection in γ-PGA production genes for soybean fermentation, and significant sequence divergence detection in quorum-sensing genes related to soybean fermentation. The assembled genome sequence revealed that the *B. subtilis *natto strain completely lacked a polyketide synthesis operon, and disrupted plipastatin production operon, and possessed previously unidentified transposases. Our natto sequence demonstrated the number and locations of insertion sequences dissimilar to *B. subtilis *Marburg 168 that possesses no typical insertion sequences. A multiple genome comparison among five closely related *Bacillus *species revealed a number of insertions and deletions but no significant rearrangements, with gene orders well conserved among the five genomes and two large syntenic segments detected.

The determined genome sequence of *B. subtilis *natto, gene predictions and annotations with the three *Bacillus *species' comparisons are available on our Natto genome browser http://natto-genome.org/.

## Methods

Several assemblers tailored for short read data such as Velvet [15], EULER [34], and SSAKE [35] have been proposed based on the de Bruijn graph. *De novo *assembly from short read data unavoidably results in a number of short scaffolds due to the presence of repeated sequences. Attempts to solve this fragmentation problem have included combining two types of short read data produced from a Roche 454 and Solexa Illumina [36], or utilizing paired-end information; however, assembly into one fully connected scaffold is difficult to achieve. On the other hand, the reference guided assembly, particularly re-sequencing, is adequate for polymorphism analysis [37] such as SNP detection among individuals in eukaryotes or very closely related bacterial strains. For the assembly of novel genomes of closely related species or distant strains, reference guided assembly cannot cover the species-specific regions divergent from a reference genome.

In order to solve these short read sequencing (SRS) problems, we have proposed a pipeline that combines *de novo *assembly and reference guided assembly to fill the gaps among *de novo *assembly scaffolds, as illustrated in Additional file 7, Figure S4. The proposed pipeline consists of four steps: (i) Short read data are mapped onto a published reference genome of closely related species, and the read data are also assembled using a *de novo *assembler. (ii) Scaffolds produced by *de novo *assembly of read data are sorted using anchors along the reference genome and then aligned to the reference genome. Anchors, which are well-conserved sequences, between each scaffold and the reference genome are calculated using Murasaki, a fast anchor finding algorithm [16]. (iii) The gaps among the sorted scaffolds are filled by aligning to the reference-guided assembly and then the scaffolds are constituted. (iv) The remaining gaps among the scaffolds are filled by a long PCR experiment and one large scaffold is finally constructed.

### Genomic DNA preparation and Illumina sequencing

BEST195 is an isolate from Miyagino-based natto [13]. *B. subtilis *Marburg 168 (1A1) was obtained from the Bacillus Genetic Stock Center (Ohio State University, Columbus, USA).

BEST195 has been used for various experiments [13,18]. A detailed SfiI physical map was constructed [18] including two plasmids, renamed pBEST195L for the large one, and pBEST195S for the smaller one. pBEST195L was similar to the self transmissible plasmid, pLS20 [20,21], and pLS195S resembled plasmids of the mobilizable plasmid family, pTA1015 [17]. In a series of plasmid preparation studies for BEST195, we fortuitously isolated a strain missing pBEST195L. Further attempts to isolate the strain missing the small pBEST195S were unsuccessful. In this study, we sequenced the genome of this strain harboring only pBEST195S. The ability to ferment boiled soybeans was not altered, and this ability was assayed according to a previously described method [13].

BEST195 genomic DNA was isolated from 5 *mL *of Luria Broth culture according to a routine biochemical isolation procedure [38] and further purified through ultracentrifugation in the presence of cesium chloride and ethidium bromide. The DNA was dissolved in 400 *μL *of Tris-EDTA (pH7.5) buffer and analyzed using pulsed-field gel electrophoresis to determine the appropriate concentration.

A single lane of an Illumina GA2 sequencer was loaded with the DNA from BEST195. The sequencer was run with 36 cycles using the standard flow cell.

### De novo assembly by Velvet and reference guided assembly by MAQ

The program used in the *de novo *assembly was Velvet 0.7 [15]. We used the following parameters on the Velvet assembler: hash_length = 23, ins_length = 110, exp_cov = 30, cov_cutoff = 10.

MAQ [14] was used for mapping the generated short reads to the reference genome of Marburg 168. We used the updated release [4] of the 168 strain genome sequence as a reference.

### Sorting de novo assembly scaffolds along reference by anchoring

Given a set of scaffolds and a reference genome sequence, the scaffolds were sorted according to anchor information along the reference genome. First, anchors, which are well-conserved sequences between each scaffold and the reference genome, were calculated using Murasaki, a fast anchor finding algorithm [16]. Murasaki enables the identification of anchors within multiple large sequences on the scale of several hundred megabases in a matter of minutes using a single CPU. Murasaki facilitates very efficient anchor generation across multiple sequences using arbitrary spaced seeds and runs on sequences several magnitudes larger than what BLASTZ can handle. Because of its unique hashing technique, Murasaki can be run in parallel to achieve arbitrarily fast wall clock times and in some cases even lower CPU times. Second, the scaffolds were sorted in the order of anchor location in the reference genome. When a scaffold contained multiple anchors, it was sorted using the longest anchor in the scaffold.

### Gene prediction and gene annotation

The gene prediction program Glimmer [39] for the prokaryote genome was applied to our Natto genome draft using the following Glimmer3 procedure:

1. ORF regions were predicted by Glimmer (ver.3.02) without a training set.

2. Based on the predicted ORFs, the ELPH program http://www.cbcb.umd.edu/software/ELPH/ was applied to calculate position weight matrix and estimate the distributions of ribosome binding sites (RBSs) and the start codons.

3. Based on the predicted distributions of RBSs and the start codons, Glimmer was reapplied to predict ORF regions more precisely.

For these predicted ORF regions, BlastN was applied to the genome sequences of *B. subtilis*, *B. licheniformis *[29], and *B. amyloliquefaciens *[28], and gene functions were annotated. Furthermore, the remaining ORFs were annotated using BlastX and the NCBI NR collection.

tRNAs were annotated using tRNAscan-SE program and rRNA was annotated using RNAmmer program.

### Synteny detection and orthologous and paralogous gene identifications

OSfinder [31] identifies syntenic segments by comparing multiple genomes. A syntenic segment is defined as a conserved segment descended from the common ancestor without rearrangements. The program takes as input anchors computed by Murasaki [16] and merges collinear anchors based on the criteria that is automatically optimized by machine learning approaches. Merged components are output as syntenic segments. As OSfinder automatically optimizes the criteria to merge anchors, syntenic segments can be identified without arbitrarily setting the criteria. Thus, rigorous results of syntenic segments can be obtained by using OSfinder.

OASYS [27] identifies one-to-one orthologs and in-paralogs [40] by comparing two genomes. When the two genomes are remotely related and the gene orders are fully disrupted, OASYS detects orthologs in the same way as the reciprocal BEST hit (RBH) method does. Otherwise, OASYS refines the results of the RBH method by combining the information of gene order conservation with the information of protein sequence similarity. Since the gene orders of Bacillus genomes have been well conserved, OASYS can accurately identify orthologs and paralogs in our analyses.

### Data deposition

All Solexa reads have been deposited in the Read Archive at DDBJ with accession number DRA000001, (ftp://ftp.ddbj.nig.ac.jp/ddbj_database/dra/DRA000001/), and the final genome sequence and annotations have been deposited in DDBJ with accession numbers AP011541 and AP011542, respectively. The determined genome sequence of *B. subtilis *natto, gene predictions and annotations with the three *Bacillus *species' comparisons are available on our Natto genome browser http://natto-genome.org/.

## Authors' contributions

YN developed genome assembly pipeline, extracted genomic DNA, carried out genome assembly, PCR experiment and gene analyses. YO carried out genome assembly, gene annotations and IS identification, and constructed Natto genome browser. TH carried out synteny detection and ortholog and paralog identifications. KP carried out scaffold sorting using Murasaki. AT and AF carried out short read sequencing by using Illumina genome analyzer. MI supervised genomic DNA extraction, carried out IS identification and gene analyses, and drafted the manuscript. YS conceived the study, carried out gene analyses, and drafted the manuscript. All authors read, revised and approved the manuscript.

## Supplementary Material

Additional file 1 **Figure S1**. Ability of *Bacillus subtilis *BEST195 to produce Natto by laboratory assay protocol. Click here for file

Additional file 2 **Data S1**. Re-sequencing results for *B. subtilis *Marburg 168. Click here for file

Additional file 3 **Figure S2**. Number of read in plipastatin biosynthesis operon region for both genomes. Click here for file

Additional file 4 **Table S1**. The list of locations of predicted transposases on BEST195 draft. Click here for file

Additional file 5 **Data S2**. The list of all the comprehensive sequence alignments for 3610 orthologous genes between *B. subtilis *natto BEST195 and Marburg 168. Click here for file

Additional file 6 **Figure S3**. The link plot of five *Bacillus *species genome comparison, and the dot plot of a pairwise comparison of orthologous genes between BEST195 and Marburg 168, *B. amyloliquefaciens*, *B. licheniformis*, and *B. pumilus *respectively. Click here for file

Additional file 7 **Figure S4**. Pipeline combining two assembly methods. Click here for file
