# Supplementary material for: Whole genome assembly of a natto production strain Bacillus subtilis natto from very short read data
Source: BMC Genomics. 2010 Apr 16;11:243. doi: 10.1186/1471-2164-11-243 (PMC2867830; doi:10.1186/1471-2164-11-243)
Supplement: Additional file 1 — Figure S1. Ability of Bacillus subtilis BEST195 to produce Natto by laboratory assay protocol. [file 1471-2164-11-243-S1.PDF]

Figure S1:
Ability of *Bacillus subtilis* BEST195 to produce Natto by laboratory assay protocol.

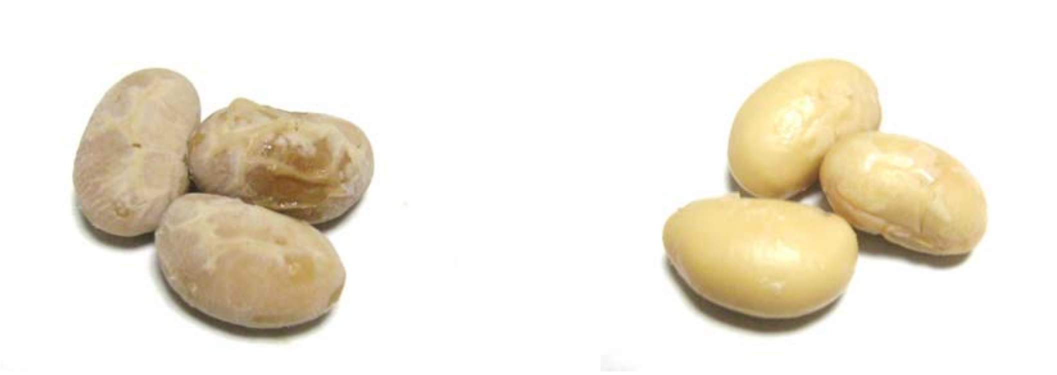

(Left) BEST195 grows on the surface of boiled soybean and produced natto-related viscous materials and fragrance. This laboratory protocol described in the reference (Itaya and Matsui, 1999) is basically identical with how to make commercial Natto, a traditional Japanese food.
(right) Marburg 168, our reference sequence strain, is incapable of producing Natto.
