# Supplementary material for: Whole genome assembly of a natto production strain Bacillus subtilis natto from very short read data
Source: BMC Genomics. 2010 Apr 16;11:243. doi: 10.1186/1471-2164-11-243 (PMC2867830; doi:10.1186/1471-2164-11-243)
Supplement: Additional file 2 — Data S1. Re-sequencing results for B. subtilis Marburg 168. [file 1471-2164-11-243-S2.PDF]

**Data S1:**

Re-sequencing results for *B. subtilis* Marburg 168.

Pair-end read data:

All reads have been deposited in the Read Archive at DDBJ with accession number DRA000002.

Assembled contigs data:

http://natto.dna.bio.keio.ac.jp/168/contig/

Consensus sequence by mapping short reads to 168 published genome using MAQ

http://natto.dna.bio.keio.ac.jp/168/consensus/
