# Supplementary material for: Whole genome assembly of a natto production strain Bacillus subtilis natto from very short read data
Source: BMC Genomics. 2010 Apr 16;11:243. doi: 10.1186/1471-2164-11-243 (PMC2867830; doi:10.1186/1471-2164-11-243)
Supplement: Additional file 3 — Figure S2. Number of read in plipastatin biosynthesis operon region for both genomes. [file 1471-2164-11-243-S3.PDF]

**Figure S2:**

Number of read in plipastatin biosynthesis operon region for both genomes.

(A) Mapping reads from BEST195 to the *pps* operon region of 168 reference genome:

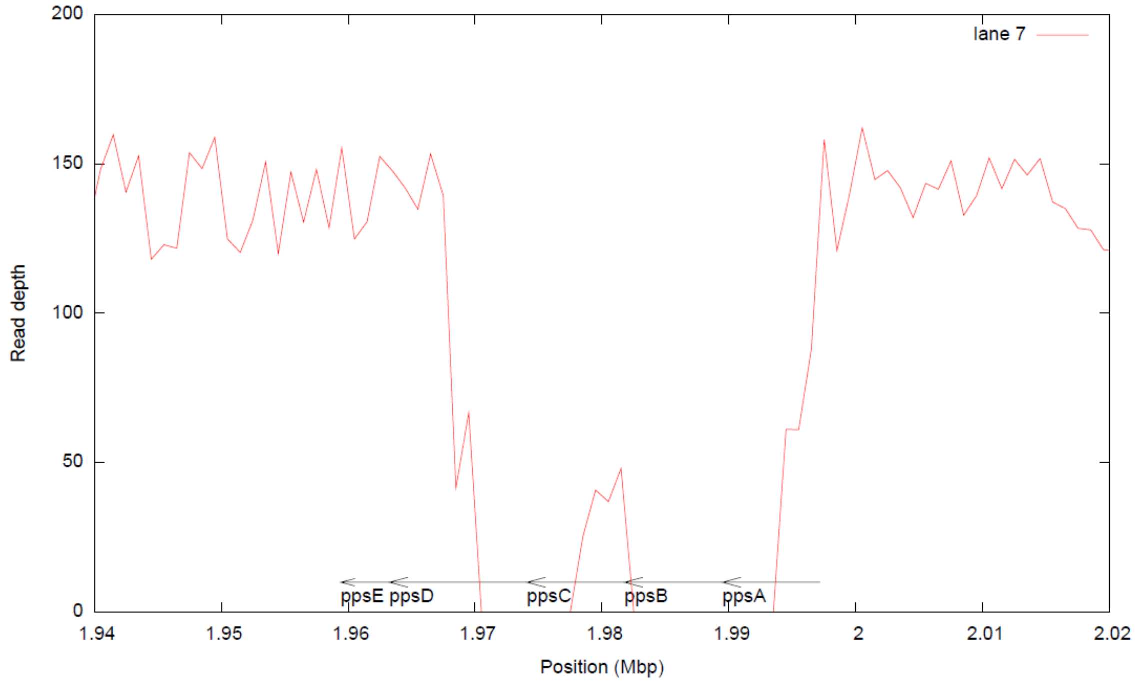


(B) Mapping reads to to the corresponding region in our BEST195 genome draft:

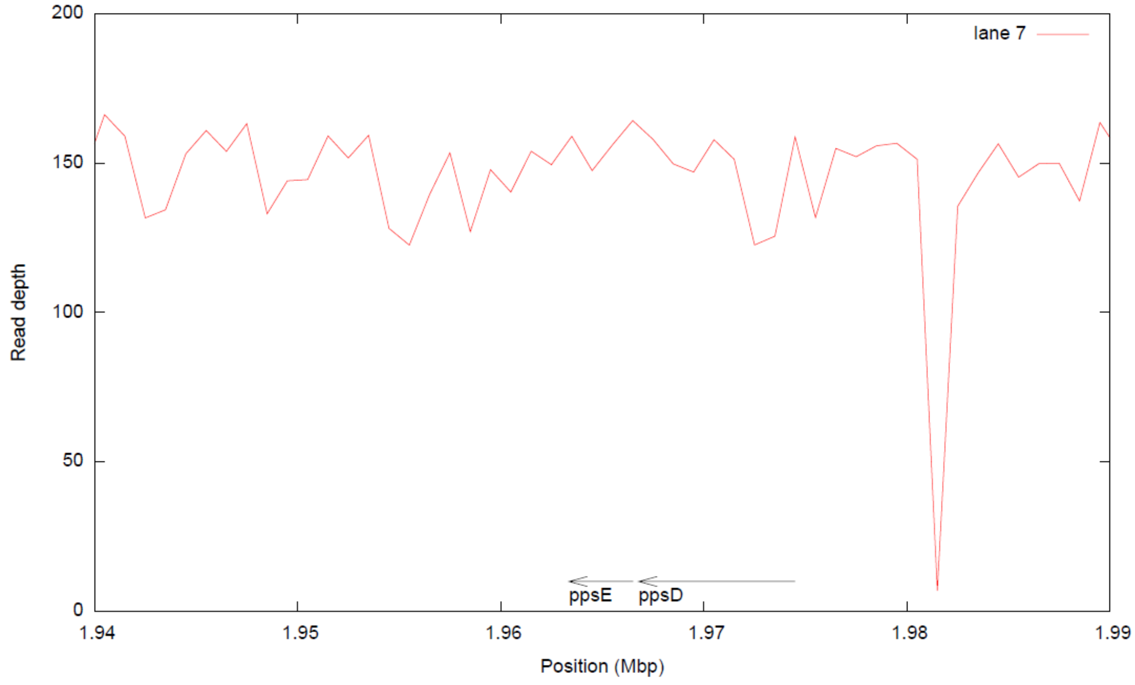

Vertical axis measures read-depth and horizontal axis enlarged the region of Marburg 168 genome in Mbp with horizontal arrows for 5 genes in the *pps* operon. Complete loss of BEST195 reads in *ppsB* and other regions with small levels of read-depth around 40-60 are apparent in (A). Small read-depth observed in *ppsA* (5'-end half), *ppsC* (5'-end half) and *ppsD* (3'-end half) are all attributed to highly repetitive sequences characteristic of those genes. Mapping reads to the corresponding region in our BEST195 genome draft gave a flat read-depth profile. Thus, only *ppsD* and *ppsE* genes are remained in BEST195.
