# Supplementary material for: Whole genome assembly of a natto production strain Bacillus subtilis natto from very short read data
Source: BMC Genomics. 2010 Apr 16;11:243. doi: 10.1186/1471-2164-11-243 (PMC2867830; doi:10.1186/1471-2164-11-243)
Supplement: Additional file 4 — Table S1. The list of locations of predicted transposases on BEST195 draft. [file 1471-2164-11-243-S4.PDF]

Table S1:

The list of locations of predicted transposases on BEST195 draft.

IS4Bsu1

BSNT_00864	539263	540387	+
BSNT_02447	1545714	1546838	+
BSNT_05424	3524302	3525426	+
BSNT_06101	3960644	3959520	-
BSNT_06124	3971732	3972856	+

IS256Bsu1

BSNT_01234	777821	778975	+
BSNT_02038	1283805	1285061	+
BSNT_02338	1475478	1474240	-
BSNT_02667	1880658	1881812	+
BSNT_02886	1896464	1897702	+
BSNT_03082	2004046	2005284	+

ISLmo1-like

orfA				orfB			
BSNT_00521	313399	313731	+	BSNT_00522	313749	314549	+
BSNT_01075	669235	668903	-	BSNT_01074	668885	668085	-
BSNT_01136	714439	714771	+	BSNT_1137	714789	715589	+
BSNT_01262	801978	802310	+	BSNT_01263	802328	803128	+
BSNT_01314	835563	835895	+	BSNT_01315	835913	836713	+
BSNT_01887	1194277	1193945	-	BSNT_01886	1193927	1193127	-
BSNT_02105	1325470	1325138	-	BSNT_02104	1325120	1324320	-
BSNT_02379	1501082	1500750	-	BSNT_02378	1500732	1500073	-
BSNT_03043	1979827	1980159	+	BSNT_03044	1980177	1980977	+
BSNT_03183	2064304	2064636	+	BSNT_03184	2064654	2065454	+
BSNT_05320	3451899	3452231	+	BSNT_05321	3452249	3453049	+

IS643-like

orfA				orfB			
BSNT_02884	1880202	1878949	-	BSNT_02883	1878952	1878194	-
BSNT_03853	2470641	2472188	+	BSNT_03854	2472185	2472943	+
BSNT_06160	4003590	4002043	-	BSNT_06159	4002046	4001288	-

ISBma2-like

BSNT_00949	596069	594717	-
BSNT_01670	1047280	1048632	+
BSNT_01786	1125606	1124254	-
BSNT_01804	1135988	1137340	+
BSNT_02272	1428586	1429938	+
BSNT_02931	1909630	1910982	+
BSNT_03543	2279177	2277825	-
BSNT_03635	2336234	2334882	-
BSNT_03954	2542066	2543418	+
BSNT_04278	2771561	2772913	+
BSNT_05336	3465355	3464003	-
BSNT_05432	3531028	3532380	+
