# Supplementary material for: Whole genome assembly of a natto production strain Bacillus subtilis natto from very short read data
Source: BMC Genomics. 2010 Apr 16;11:243. doi: 10.1186/1471-2164-11-243 (PMC2867830; doi:10.1186/1471-2164-11-243)
Supplement: Additional file 5 — Data S2. The list of all the comprehensive sequence alignments for 3610 orthologous genes between B. subtilis natto BEST195 and Marburg 168. [file 1471-2164-11-243-S5.PDF]

Data S2:

The list of comprehensive sequence alignments for 3610 orthologous genes between *B. subtilis* natto BEST195 and Marburg 168.

http://natto.dna.bio.keio.ac.jp/alignment
