# Supplementary material for: Whole genome assembly of a natto production strain Bacillus subtilis natto from very short read data
Source: BMC Genomics. 2010 Apr 16;11:243. doi: 10.1186/1471-2164-11-243 (PMC2867830; doi:10.1186/1471-2164-11-243)

Figure S3:

The link plot of five *Bacillus* species genome comparison, and the dot plot of a pairwise comparison of orthologous genes between BEST195 and Marburg 168, *B.amyloliquefaciens*, *B.licheniformis*, and *B.pumilus* respectively.

(A) The link plot of five *Bacillus* species genome comparison: Marburg 168, *B.amyloliquefaciens*, BEST195, *B.licheniformis*, and *B.pumilus* from the top.

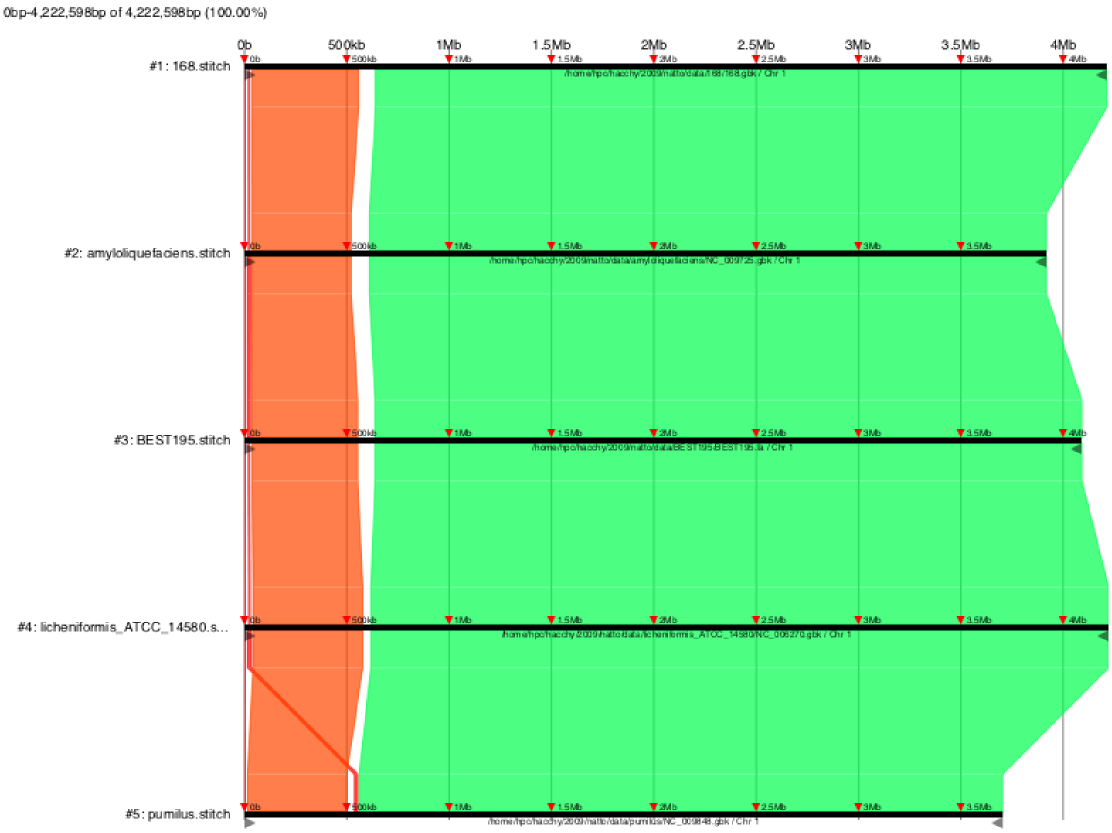

(B) The dot plot of a pairwise comparison of orthologous genes between BEST195 (horizontal axis) and Marburg 168 (vertical axis):

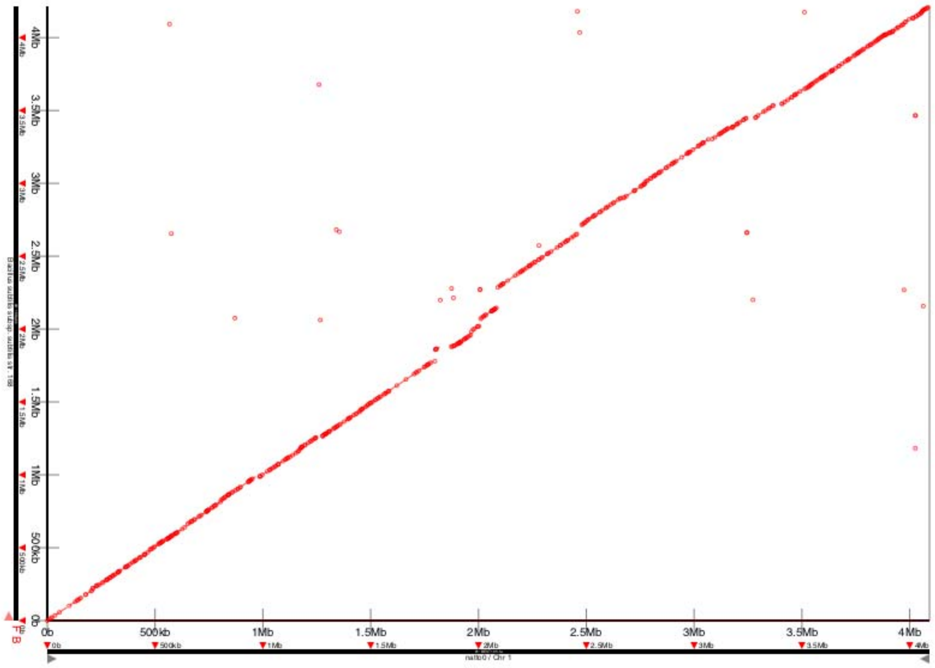

(C) The dot plot between BEST195 and *B.amyloliquefaciens* (vertical axis):

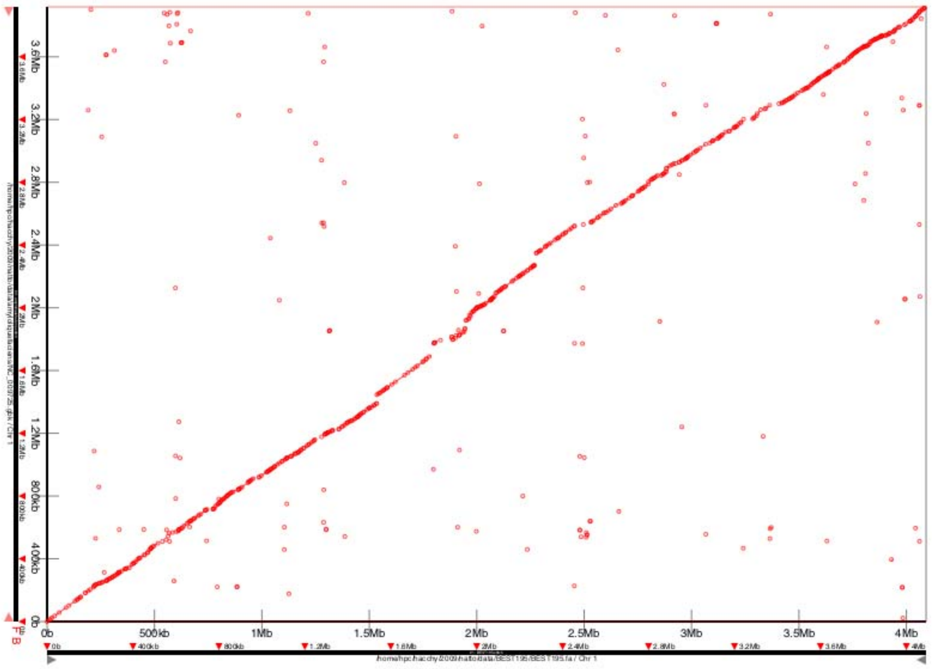

(D) The dot plot between BEST195 and *B.licheniformis* (vertical axis):

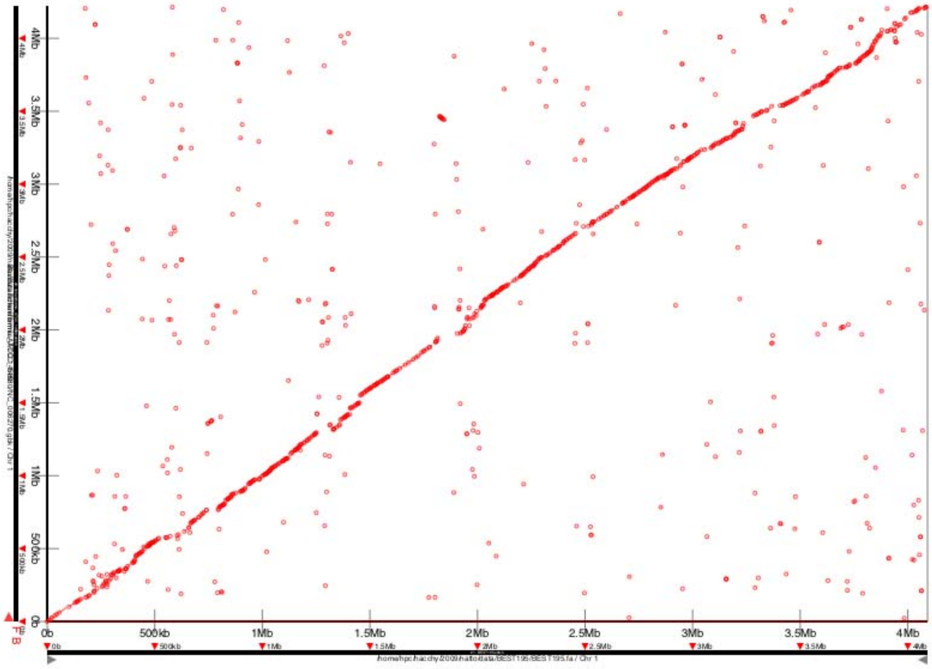

(E) The dot plot between BEST195 and *B.pumilus* (vertical axis):

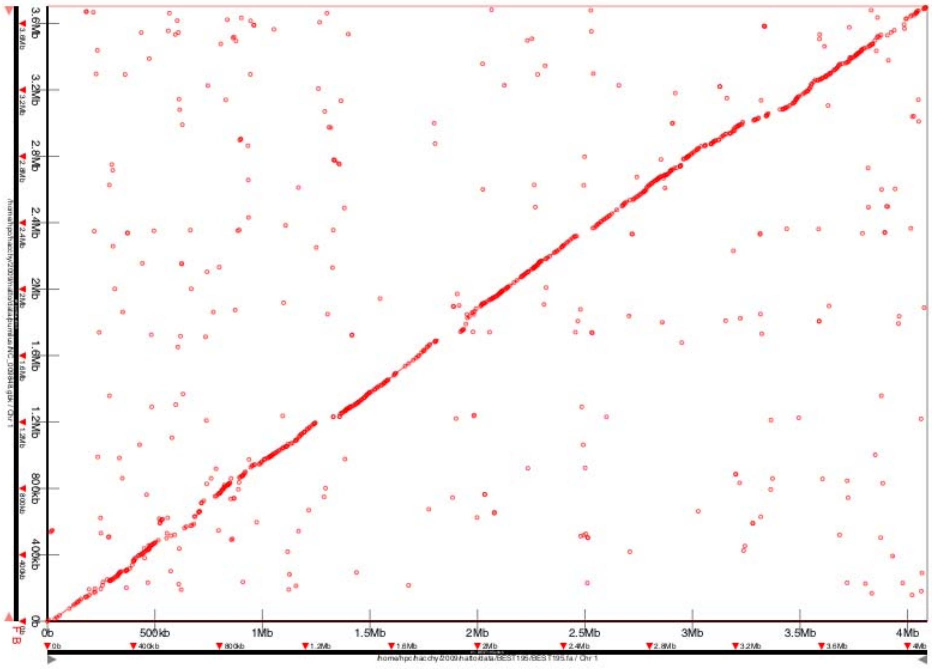

Supplement: Additional file 6 — Figure S3. The link plot of five Bacillus species genome comparison, and the dot plot of a pairwise comparison of orthologous genes between BEST195 and Marburg 168, B. amyloliquefaciens, B. licheniformis, and B. pumilus respectively. [file 1471-2164-11-243-S6.PDF]
