# Supplementary material for: Whole genome assembly of a natto production strain Bacillus subtilis natto from very short read data
Source: BMC Genomics. 2010 Apr 16;11:243. doi: 10.1186/1471-2164-11-243 (PMC2867830; doi:10.1186/1471-2164-11-243)
Supplement: Additional file 7 — Figure S4. Pipeline combining two assembly methods. [file 1471-2164-11-243-S7.PDF]

Figure S4:

Pipeline combining two assembly methods.

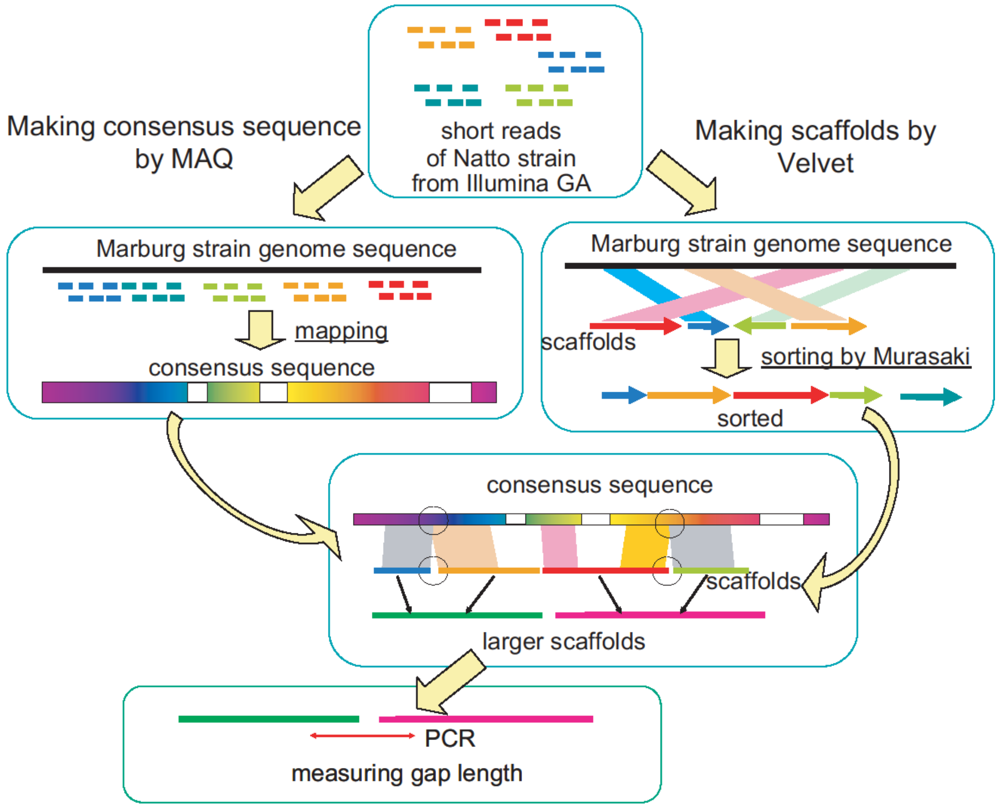

We propose a pipeline that combines de novo assembly and reference guided assembly to evade substantial fragmention in assembling from short read data.

The proposed pipeline consists of four steps:

(i) Short read data are mapped onto a published reference genome of closely related species, and the read data are also assembled using a de novo assembler.

(ii) Scaffolds produced by de novo assembly of read data are sorted using anchors along the reference genome and then aligned to the reference genome. Anchors, which are well-conserved sequences, between each scaffold and the reference genome are calculated using Murasaki, a fast anchor finding algorithm.

(iii) The gaps among the sorted scaffolds are filled by aligning to the reference-guided assembly and then the scaffolds are constituted.

(iv) The remaining gaps among the scaffolds are filled through a long PCR experiment and one large scaffold is finally constructed.
